# Hernia

**Published:** 1897-04

**Authors:** 


### HERNIA.

George Heaton, M. D., states that in his experience, hernia becomes less frequent in children year by year, up to the age of twelve or thirteen, and believes that this is due to the tendency toward spontaneous cure in the very young. He also thinks that in making a prognosis, the following are the chief facts to be considered:

Age: The younger the patient and the earlier the treatment is begun, the better the prospect of ultimate cure.

The character of the hernia: Umbilical hernia is usually cured by proper attention, while femoral hernia is rarely cured by truss, and seldom disappears spontaneously: inguinal hernia may partake of either characteristics.

Sex: Inguinal hernia in girls may usually be cured by truss.

Due regard must be had to avoid any cause of irritation or straining, such as diarrhœa, colic, bronchitis, threadworms, etc.; and should operations appear to be necessary, it is well to take into consideration the age, the financial condition of the parents, and the irreducibility of the hernia—as the younger the child the better the chance of a cure without operation; and if the parents can not afford a long course of treatment, an early operation is advisable; while if a suitable truss can not be properly applied, or if the hernia is complicated by testis in the inguinal canal, an operation is necessary.

Dr. Heaton further says that he goes no further than to cut away the sac after ligaturing its neck, and does not even suture the pillars of the external ring. In cases of congenital hernia he forms a tunica vaginalis by closing the lowest portion of the sac by means of a continuous suture. With the use of antiseptics such operations are attended by little risk, and the patient should be kept in bed for five or six days to permit the parts to thoroughly consolidate.

Twenty-three such operations have been performed by him, resulting in twenty perfect cures, one relapse soon after operation, and two relapses some months afterward—a very good record.

He also says that for children under two years of age, the best form of truss is one of soft worsted, of which several should be kept on hand and changed as often as desirable. Above that age, a well-made spring truss, covered with leather, should be used, and he advises that it be always applied by the physician himself.

Such a truss should be constantly worn, if a cure is to be expected, and the parts should be occasionally bathed with weak alcohol and water to keep the skin from chafing. Care must also be constantly exercised not to permit the hernial contents from descending.—*A. M. S. Bulletin.*

